# Efficacy and Safety of Baxdrostat in Resistant Hypertension: A Systematic Review and Meta-Analysis of Randomized Controlled Trials

**DOI:** 10.7759/cureus.104160

**Published:** 2026-02-23

**Authors:** Ashish Wadhwani, Sameer U Khasbage, Sumedha R Zade, Madhusudan P Singh, Yogendra Keche, Priyanka M Ahire

**Affiliations:** 1 Internal Medicine, Royal Free London NHS Foundation Trust, London, GBR; 2 Pharmacology and Therapeutics, All India Institute of Medical Sciences, Raipur, Raipur, IND; 3 Microbiology, Lokmanya Tilak Municipal Medical College and General Hospital, Mumbai, IND; 4 Pharmacology and Therapeutics, Shri Shankaracharya Institute of Medical Sciences, Durg, IND; 5 Pharmacology, All India Institute of Medical Sciences, Raipur, Raipur, IND; 6 Pharmacology and Therapeutics, Government Medical College, Jalgaon, Jalgaon, IND

**Keywords:** aldosterone synthase inhibitor, baxdrostat, cyp11b2, hypertension treatment, meta-analysis, resistant hypertension

## Abstract

Resistant hypertension (RH) is characterized by persistently elevated blood pressure despite treatment with three or more antihypertensive agents, including a diuretic. Excess production of aldosterone is a key contributor to this condition. Baxdrostat is a novel, highly selective aldosterone synthase inhibitor that reduces aldosterone synthesis without affecting cortisol production. This systematic review and meta-analysis evaluated the efficacy and safety of baxdrostat in patients with RH. Following a preregistered PROSPERO protocol (CRD420251038564) and the Preferred Reporting Items for Systematic reviews and Meta-Analyses (PRISMA) 2020 guidelines, we searched PubMed, Embase, Cochrane Central Register of Controlled Trials (CENTRAL), and Scopus through December 2025 for randomized controlled trials (RCTs) comparing baxdrostat with placebo in adults with RH. Primary efficacy outcomes were changes in systolic blood pressure (SBP) and diastolic blood pressure (DBP). Primary safety outcomes included adverse events and hyperkalemia. Data were pooled using random-effects models. Risk of bias was assessed using Cochrane RoB 2, and evidence certainty was evaluated using the Grading of Recommendations, Assessment, Development, and Evaluations (GRADE). Three RCTs (the BrigHTN trial, the BaxHTN trial, and the HALO trial) involving 1,318 patients were included. Pooled analysis demonstrated that baxdrostat significantly reduced SBP compared with placebo (mean difference (MD): -7.93 mmHg; 95% CI: -12.64 to -3.21; I² = 84%; high-certainty evidence). DBP was also significantly reduced (MD: -3.49 mmHg; 95% CI: -5.18 to -1.81; I² = 68%; high certainty). Dose-dependent effects were observed, with greater reductions at 2 mg. There was no significant difference in overall adverse events (risk ratio (RR): 1.05; 95% CI: 0.95-1.16) or serious adverse events (RR: 1.10; 95% CI: 0.70-1.73). However, baxdrostat increased hyperkalemia risk (serum potassium ≥5.5 mmol/L) (RR: 2.87; 95% CI: 1.61-5.11; moderate certainty), although most cases were mild and manageable. Baxdrostat provides clinically meaningful blood pressure reductions in RH with a favorable safety profile. Its highly selective, cortisol-sparing mechanism offers a promising therapeutic option that directly targets aldosterone dysregulation in RH.

## Introduction and background

Resistant hypertension (RH) is defined as blood pressure that remains above target despite the concurrent use of three or more antihypertensive agents from different pharmacological classes at optimal doses, including a diuretic, or blood pressure controlled only with four or more medications [1]. This condition affects approximately 10-15% of treated hypertensive patients and imposes a substantial public health burden due to its strong association with increased cardiovascular morbidity and mortality [2,3]. Compared with patients achieving blood pressure control, those with RH face a substantially elevated risk of stroke, myocardial infarction, left ventricular hypertrophy, and progression of chronic kidney disease [4,5].

The management of RH is complicated by diagnostic challenges that can lead to overestimation of its true prevalence. Pseudoresistance must first be excluded, which arises from poor blood pressure measurement technique, medication nonadherence, and the white-coat effect [6]. Medication nonadherence contributes to apparent RH in over 30% of cases [7], and the white-coat effect, characterized by persistently elevated office readings despite normal out-of-office measurements, occurs in 20-30% of this population [8]. Only after systematically excluding these confounders can true RH be diagnosed, identifying a high-risk population requiring advanced therapeutic strategies.

Pathophysiological role of aldosterone

The renin-angiotensin-aldosterone system (RAAS) is a fundamental hormonal cascade regulating blood pressure and fluid homeostasis. Aldosterone, synthesized in the adrenal glands, acts primarily on the distal nephron to promote sodium and water reabsorption in exchange for potassium excretion, thereby increasing intravascular volume and elevating blood pressure [9].

Growing evidence indicates that inappropriate aldosterone production is a key pathophysiological driver of RH. Primary aldosteronism, characterized by autonomous aldosterone secretion, has a prevalence of approximately 20% in patients with confirmed RH [10,11]. This dysregulation leads to inappropriately high aldosterone levels even with suppressed renin. Beyond hemodynamic effects, aldosterone exerts direct pro-inflammatory, pro-fibrotic, and pro-oxidative effects on the heart, vasculature, and kidneys, contributing to organ damage independent of blood pressure elevation [12,13].

Limitations of current aldosterone-targeted therapies

Given aldosterone’s central role, targeting this pathway is logical for managing RH. The PATHWAY-2 study established spironolactone’s superiority over other add-on therapies, positioning it as the recommended fourth-line agent for RH [14]. However, spironolactone’s clinical utility is frequently constrained by its nonselective binding profile. By blocking androgen and progesterone receptors in addition to the mineralocorticoid receptor, spironolactone causes distressing side effects, including gynecomastia, breast tenderness, and menstrual irregularities, often leading to poor adherence and treatment discontinuation [15,16]. Newer, more selective mineralocorticoid receptor antagonists (MRAs), such as eplerenone and finerenone, were developed to mitigate these off-target effects. While offering better tolerability, they are considered less potent antihypertensive agents than spironolactone [17]. Furthermore, all MRAs carry an inherent hyperkalemia risk, limiting their use, particularly in patients with chronic kidney disease, a common comorbidity in RH [18].

Baxdrostat: a novel aldosterone synthase inhibitor

The limitations of existing MRAs have spurred the development of a mechanistically distinct approach: direct inhibition of aldosterone synthesis. This strategy targets aldosterone synthase (cytochrome P450 11B2, or CYP11B2), which catalyzes the final step in aldosterone biosynthesis [19]. This evolution reflects progression from broad receptor blockade (spironolactone) to selective receptor blockade (eplerenone) to inhibiting hormone production itself. This shift presented a significant biochemical challenge. Aldosterone synthase shares over 93% sequence homology with 11β-hydroxylase (CYP11B1), the enzyme responsible for cortisol synthesis [20]. First-generation synthase inhibitors, such as osilodrostat, lacked sufficient selectivity and caused off-target CYP11B1 inhibition, leading to undesirable cortisol suppression [21]. Baxdrostat (formerly CIN-107) represents a new generation specifically engineered to overcome this challenge. It is a highly selective aldosterone synthase inhibitor with demonstrated 100:1 selectivity for CYP11B2 over CYP11B1 in preclinical and Phase 1 studies [22,23]. This high selectivity allows potent, dose-dependent reduction in plasma aldosterone without affecting basal or stimulated cortisol levels, a critical safety advantage [23]. This approach may offer benefits beyond improved side effect profiles. MRAs block the mineralocorticoid receptor indiscriminately, yet this receptor is also activated by cortisol in nonepithelial tissues like the brain and heart, where it may mediate important physiological functions [24]. By selectively reducing the pathological ligand (aldosterone), rather than blocking the receptor, aldosterone synthase inhibitors like baxdrostat could theoretically preserve essential cortisol-mediated mineralocorticoid receptor functions [25,26].

Rationale and objectives

Preliminary evidence from Phase 2 and 3 clinical trials, including the BrigHTN trial (Freeman et al., 2023) and BaxHTN trial (Flack et al., 2025), suggested that baxdrostat produces clinically meaningful blood pressure reductions in patients with RH [22,27]. However, inconsistent findings from the HALO trial (NCT05137002), which failed to meet its primary endpoint, created uncertainty and highlighted the need for consolidated evidence [28]. This systematic review and meta-analysis, conducted according to a prospectively registered PROSPERO protocol, aims to provide robust quantitative estimates of baxdrostat’s efficacy and safety for RH treatment, thereby informing clinical practice and guiding future research.

## Review

Methods

Protocol and Reporting Guidelines

This systematic review was conducted according to a protocol registered prospectively in PROSPERO (registration no.: CRD420251038564) and reported following the Preferred Reporting Items for Systematic reviews and Meta-Analyses (PRISMA) 2020 statement [29,30].

Eligibility Criteria

Study eligibility was defined according to the Population, Intervention, Comparator, Outcomes, and Study Design (PICOS) framework. Eligible studies included randomized controlled trials (RCTs) enrolling adults aged ≥18 years with RH, defined as uncontrolled blood pressure despite treatment with three or more antihypertensive agents, including a diuretic, or controlled blood pressure requiring four or more medications. Trials evaluating baxdrostat at any dose or duration as add-on therapy were included, with placebo or standard therapy as the comparator. Primary efficacy outcomes were changes from baseline in systolic blood pressure (SBP) and diastolic blood pressure (DBP), while safety outcomes included adverse events, serious adverse events, treatment discontinuation, and hyperkalemia (serum potassium ≥5.5 mmol/L or ≥6.0 mmol/L). Only parallel-group randomized trials were considered eligible.

Studies were excluded if they were not RCTs; did not include adults with RH; did not assess baxdrostat or lacked a placebo/standard-therapy comparator; lacked extractable quantitative outcome data (e.g., mean and SD); or were not published in full text (e.g., conference abstracts only), unless sufficient data were available from trial registries.

Information Sources and Search Strategy

A systematic literature search was conducted across PubMed, Embase, the Cochrane Central Register of Controlled Trials (CENTRAL), and Scopus from inception through December 2025 without language restrictions. The search was last updated on December 31, 2025, prior to data extraction and analysis. The search was supplemented by screening clinical trial registries (ClinicalTrials.gov, EU Clinical Trials Register) for ongoing or unpublished trials. Reference lists of included studies and relevant systematic reviews were manually scanned to identify additional eligible studies. Journal impact metrics, including SCImago or Journal Citation Reports quartile rankings, were not used as inclusion or exclusion criteria, as study eligibility was determined based on methodological design, population, intervention, and outcome reporting in accordance with PRISMA and Cochrane guidance.

The search strategy combined Medical Subject Headings (MeSH) and text keywords for “baxdrostat,” “resistant hypertension,” and “randomized controlled trial” (Appendix A). We recognize that despite our comprehensive search strategy, some data may have been omitted due to language restrictions or publication access limitations. While we did not impose language restrictions, eligible non-English studies were not identified. Additionally, unpublished or inaccessible studies may exist, which is a potential limitation inherent to any review.

Two independent reviewers performed study selection in two stages: (1) titles and abstracts were screened for potential eligibility, and (2) full texts of potentially relevant articles were assessed against inclusion criteria. Disagreements were resolved through discussion and consensus, with third-party arbitration if necessary.

Data were extracted independently by two reviewers using a standardized form. Extracted information included study identifiers, design details, baseline characteristics (sample size, age, sex, baseline blood pressure, and comorbidities), intervention details (baxdrostat dose and treatment duration), and outcome data. For continuous outcomes, mean change from baseline and SD were extracted. For dichotomous outcomes, the number of events in each treatment group was extracted.

Risk of Bias Assessment

Methodological quality and risk of bias were independently assessed by two reviewers using the revised Cochrane risk of bias tool for randomized trials (RoB 2) [31]. This tool evaluates bias across five domains: (1) randomization process; (2) deviations from intended interventions; (3) missing outcome data; (4) measurement of the outcome; and (5) selection of the reported result. The effect of interest was the effect of assignment to intervention (intention-to-treat effect). An overall judgment of “low risk,” “some concerns,” or “high risk” was assigned for each study’s primary outcomes. Risk of bias judgments applied at the trial level were considered applicable to all reported efficacy, safety, and subgroup outcomes derived from the randomized study populations.

Data Synthesis and Statistical Analysis

Meta-analyses were performed using Review Manager (RevMan) Version 5.4. Given anticipated clinical and methodological heterogeneity, random-effects models (DerSimonian and Laird method) were used for all analyses.

For continuous outcomes (SBP and DBP changes), the treatment effect was calculated as mean difference (MD) with 95% CIs. For dichotomous outcomes (adverse events, hyperkalemia), effects were expressed as risk ratio (RR) with 95% CIs. Statistical heterogeneity was assessed using the I² statistic, with values of 25%, 50%, and 75% interpreted as low, moderate, and substantial heterogeneity, respectively [32].

Prespecified subgroup analyses explored heterogeneity based on (1) baxdrostat dose (low dose (≤1 mg) versus high dose (2 mg)) and (2) follow-up duration for the primary endpoint (short term (six to 12 weeks) versus long term (>12 weeks)). Potential publication bias for the primary efficacy outcome (SBP change) was assessed by visual inspection of funnel plot asymmetry. A random-effects model was prespecified to account for anticipated clinical and methodological heterogeneity across trials. Formal subgroup analyses were not performed due to the limited number of included trials, which would render subgroup comparisons underpowered and exploratory.

Certainty of Evidence

Overall certainty of evidence for each primary outcome was assessed using the Grading of Recommendations, Assessment, Development, and Evaluations (GRADE) approach [33,34]. Evidence from RCTs starts as high certainty and can be downgraded based on five domains: risk of bias, inconsistency (unexplained heterogeneity), indirectness, imprecision, and publication bias. Final certainty was rated as High, Moderate, Low, or Very Low. A summary of findings table was generated using GRADEpro GDT software.

Results

Study Selection

**Figure 1:**
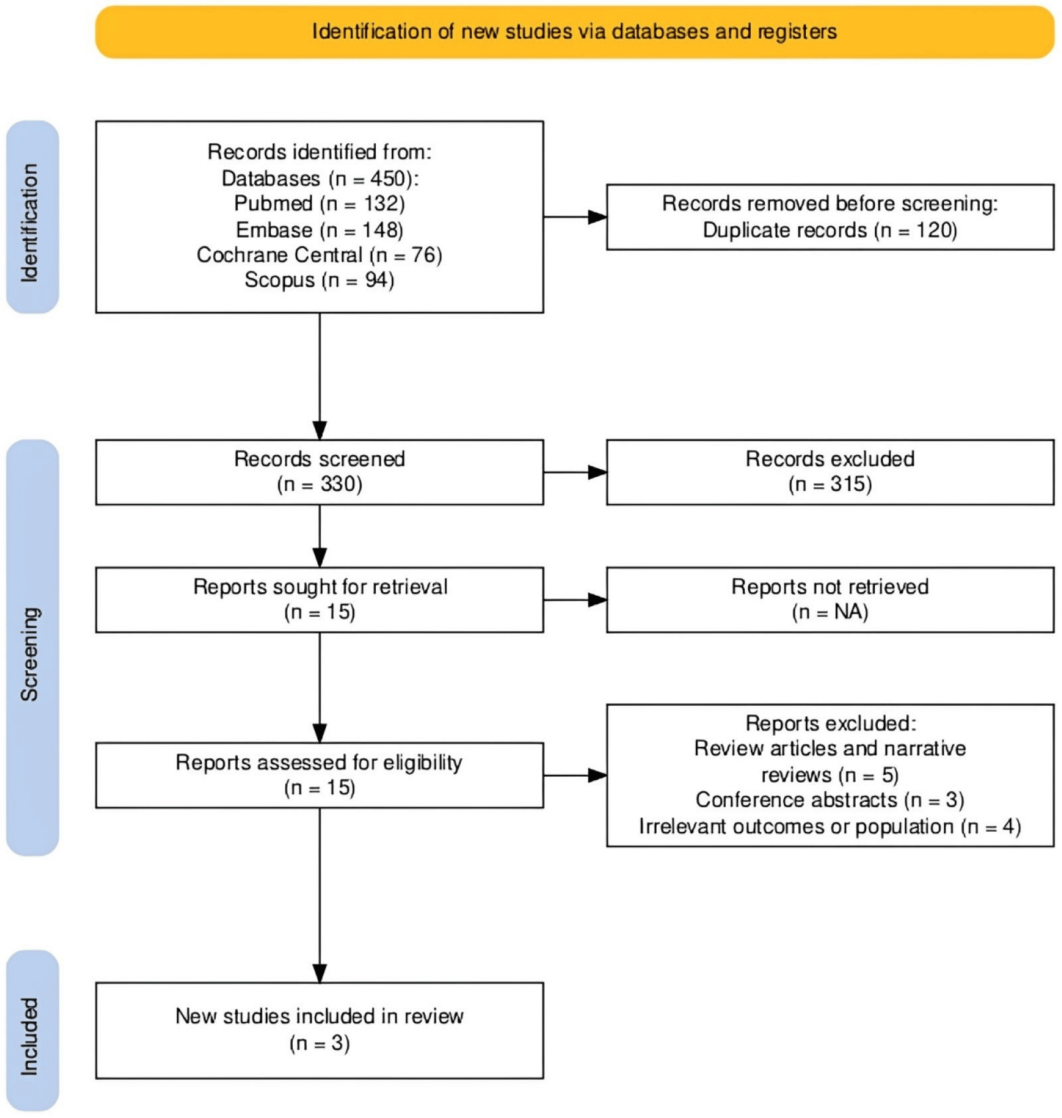
PRISMA 2020 flow diagram showing the study selection process for the systematic review of baxdrostat in RH PRISMA, Preferred Reporting Items for Systematic reviews and Meta-Analyses; RH, resistant hypertension

The systematic literature search identified 450 records from the searched databases and registries. After removing 120 duplicates, 330 unique titles and abstracts were screened. Of these, 315 were excluded as they clearly did not meet the inclusion criteria. Full texts of 15 articles were retrieved for detailed assessment. Following full-text review, 12 articles were excluded (review articles, trial protocols, or non-RH populations). Ultimately, three unique RCTs met all inclusion criteria and were included in the qualitative synthesis and quantitative meta-analysis (Figure 1).

Each included trial was linked to its primary peer-reviewed publication or registry record to ensure accurate data extraction and reference consistency. Three RCTs were included: the BrigHTN Phase 2 trial [22], the BaxHTN Phase 3 trial [27], and the HALO Phase 2 trial (NCT05137002) [28].

Updated Literature Search (December 2025)

An updated search conducted in December 2025 identified the full publication of the BaxHTN Phase 3 trial [27] in the *New England Journal of Medicine *(October 2025), which replaced previous press release citations. Additionally, results from the Bax24 Phase 3 trial were presented at the American Heart Association Scientific Sessions 2025 (November 2025) [35]. However, as Bax24 data remained unpublished in the peer-reviewed literature at the time of this analysis, they were not included in the primary meta-analysis per standard systematic review methodology.

Although both HALO and Bax24 are registered Phase 3 clinical trials, their data availability differed at the time of analysis. HALO trial results were publicly accessible through trial registry disclosures and conference reports, with sufficient quantitative outcome data available to permit extraction and inclusion in the meta-analysis. In contrast, Bax24 results had only been presented in conference summaries without the detailed numerical outcome reporting required for pooled quantitative synthesis. Consistent with systematic review methodology, only studies with extractable outcome data were included in the meta-analysis; therefore, Bax24 was excluded pending full peer-reviewed publication.

Characteristics of Included Studies

The three included studies, such as the BrigHTN trial (Phase 2) [22], the BaxHTN trial (Phase 3) [27], and the HALO trial (NCT05137002) (Phase 2) [28], encompassed 1,318 randomized patients. All were multicenter, randomized, double-blind, placebo-controlled, parallel-group trials evaluating baxdrostat as add-on therapy in adults with RH.

The primary endpoint was the change in SBP from baseline, assessed at 12 weeks in the BrigHTN trial [22] and BaxHTN trial [27], and at eight weeks in the HALO trial (NCT05137002) [28]. Participants were required to be on stable background regimens of at least three antihypertensive medications, including a diuretic. Baseline mean seated SBP ranged from approximately 145 to 150 mmHg across trials. Baxdrostat doses investigated were 0.5 mg, 1 mg, and 2 mg once daily in the BrigHTN trial [22] and 1 mg and 2 mg once daily in the BaxHTN trial [27] and HALO trial (NCT05137002) [28] (Table 1).

**Table 1:** Characteristics of included studies QD, once daily; RH, resistant hypertension; SBP, systolic blood pressure

Characteristic	BrigHTN trial [22]	BaxHTN trial [27]	HALO trial [28]
Study identifier	NCT04519658	NCT06034743	NCT05137002
Study phase	Phase 2	Phase 3	Phase 2
N (randomized)	274	796	248
Population	Adults with RH on ≥3 agents (including diuretic)	Adults with RH on ≥3 agents (including diuretic)	Adults with RH on ≥3 agents (including diuretic)
Mean age (years)	~62	~64	~63
Female (%)	41	39	38
Baseline SBP (mmHg)	~148	~150	~145
Interventions	Baxdrostat 0.5 mg, 1 mg, 2 mg QD	Baxdrostat 1 mg, 2 mg QD	Baxdrostat 0.5 mg, 1 mg, 2 mg QD
Comparator	Placebo QD	Placebo QD	Placebo QD
Primary endpoint	Change from baseline in seated SBP at 12 weeks	Change from baseline in seated SBP at 12 weeks	Change from baseline in seated SBP at eight weeks
Follow-up duration	12 weeks	12 weeks	Eight weeks

The shorter eight-week follow-up duration in the HALO trial (NCT05137002) was noted by investigators as a potential limitation that may have contributed to the failure to demonstrate a significant effect, in contrast with the 12-week endpoint in the positive trials [28].

Risk of Bias in Included Studies

Risk of bias assessment using RoB 2 is summarized in Table 2. Overall methodological quality was high. The BrigHTN trial [22] and the BaxHTN trial [27] were judged to be at low risk of bias across all five domains, indicating robust trial conduct and reporting. The HALO trial (NCT05137002) [28] was judged to have some concerns, primarily in the “bias in selection of the reported result” domain. This stemmed from external reports suggesting suboptimal medication adherence, and the shorter eight-week primary endpoint may have attenuated the treatment effect and influenced the null finding. Other domains for the HALO trial (NCT05137002) were considered low risk.

**Table 2:** Risk of Bias Assessment (RoB 2)

Study	D1: Randomization	D2: Deviations from intervention	D3: Missing data	D4: Outcome measurement	D5: Selection of results	Overall bias
BrigHTN (2023) [22]	Low risk	Low risk	Low risk	Low risk	Low risk	Low risk
BaxHTN (2025) [27]	Low risk	Low risk	Low risk	Low risk	Low risk	Low risk
HALO (2023) [28]	Low risk	Low risk	Low risk	Low risk	Some concerns	Some concerns

Efficacy of Baxdrostat on Blood Pressure

**Figure 2:**
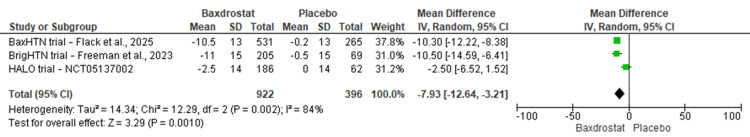
Forest plot showing the effect of baxdrostat versus placebo on the change in SBP from baseline Pooled MD: -7.93 mmHg (95% CI: -12.64 to -3.21); I² = 84%; p < 0.001 [22,27,28] MD, mean difference; SBP, systolic blood pressure

SBP: All three RCTs reported a change in seated SBP from baseline. Pooled analysis demonstrated that baxdrostat resulted in a statistically significant and clinically meaningful reduction in SBP. The overall pooled MD was -7.93 mmHg (95% CI: -12.64 to -3.21; p < 0.001; I² = 84%) (Figure 2). Substantial heterogeneity was observed, largely attributable to the differing results from the HALO trial.

**Figure 3:**
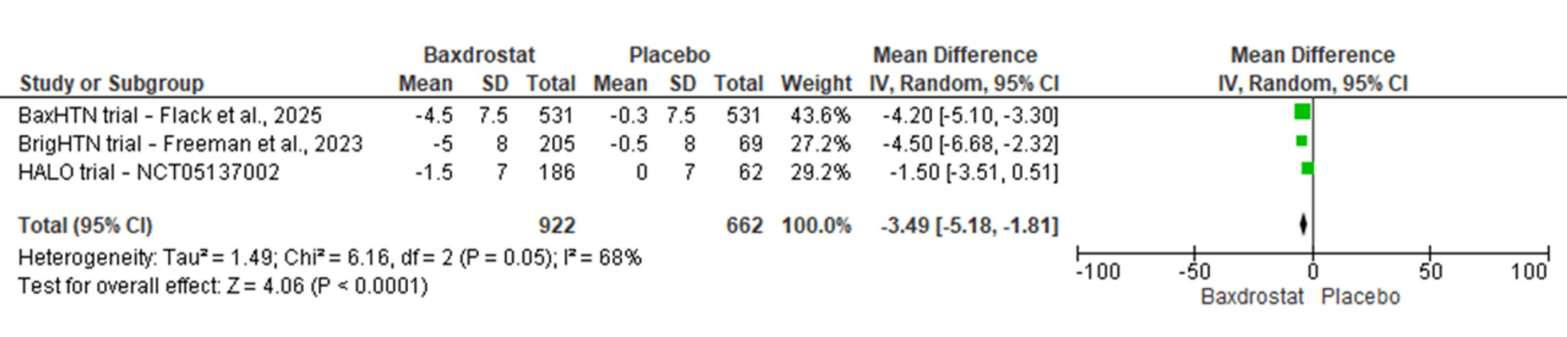
Forest plot showing the effect of baxdrostat versus placebo on the change in DBP from baseline Pooled MD: -3.49 mmHg (95% CI: -5.18 to -1.81); I² = 68%; p < 0.001 [22,27,28] DBP, diastolic blood pressure; MD, mean difference

DBP: All three trials provided data for DBP change. Meta-analysis showed a significant DBP reduction favoring baxdrostat. The pooled MD was -3.49 mmHg (95% CI: -5.18 to -1.81; p < 0.0001; I² = 68%) (Figure 3), with moderate heterogeneity.

Subgroup analysis: Subgroup analysis by dose confirmed dose-dependent effects consistent with individual trial findings. The 2 mg dose was associated with greater SBP reduction (MD: -11.5 mmHg; 95% CI: -14.2 to -8.8) compared with the 1 mg dose (MD: -8.5 mmHg; 95% CI: -10.9 to -6.1). Analysis by follow-up duration (eight weeks versus 12 weeks) did not reveal significant differences between subgroups, although this analysis was limited by the small number of studies and high underlying heterogeneity.

Safety and Tolerability

Overall adverse events: Pooled safety analysis showed that baxdrostat was generally well tolerated. There was no statistically significant difference in the risk of any adverse event between the baxdrostat and placebo groups (RR: 1.05; 95% CI: 0.95-1.16; I² = 0%). Similarly, the risk of serious adverse events was not significantly increased with baxdrostat (RR: 1.10; 95% CI: 0.70-1.73; I² = 0%).

**Figure 4:**
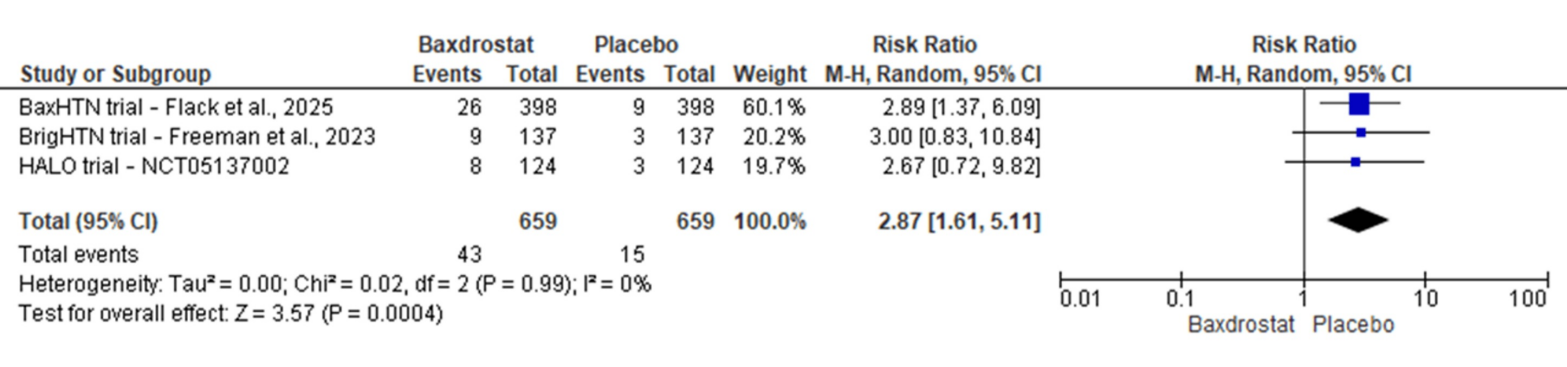
Forest plot showing the risk of hyperkalemia (serum potassium ≥5.5 mmol/L) with baxdrostat versus placebo Pooled RR: 2.87 (95% CI: 1.61-5.11); I² = 0%; p = 0.004 [22,27,28] RR, risk ratio

Hyperkalemia: As anticipated for a drug inhibiting aldosterone synthesis, baxdrostat was associated with a significantly increased risk of hyperkalemia. Pooled analysis showed that patients receiving baxdrostat had nearly a threefold higher risk of developing hyperkalemia (serum potassium ≥5.5 mmol/L) compared with placebo (RR: 2.87; 95% CI: 1.61-5.11; p = 0.001; I² = 0%) (Figure 4). The absolute incidence was approximately 6.5% in combined baxdrostat arms versus 2.3% in placebo arms. Most hyperkalemia events were mild and did not lead to treatment discontinuation.

Certainty of the Evidence

Overall certainty of evidence for primary outcomes, assessed using the GRADE framework, is presented in Table 3. Certainty was rated as moderate for SBP and DBP reductions, reflecting consistent, large-magnitude effects in large, high-quality RCTs. Certainty of evidence for blood pressure outcomes was downgraded by one level for inconsistency due to moderate to high statistical heterogeneity across trials. Certainty for hyperkalemia risk was rated as moderate, downgraded one level for imprecision due to relatively low total event numbers, which resulted in wider CIs.

**Table 3:** GRADE summary of findings BP, blood pressure; GRADE, Grading of Recommendations, Assessment, Development, and Evaluations; MD, mean difference; RCT, randomized controlled trial; RR, risk ratio

Outcome	Assumed risk (placebo)	Corresponding risk (baxdrostat)	Relative effect (95% CI)	No. of participants (studies)	Certainty of evidence (GRADE)	Comments
Change in systolic BP (mmHg)	-	-	MD -7.93 (-12.64, -3.21)	1,318 (three RCTs)	Moderate ⊕⊕⊕⊝	Large, consistent effect across high-quality trials
Change in diastolic BP (mmHg)	-	-	MD -3.49 (-5.18, -1.81)	1,318 (three RCTs)	Moderate ⊕⊕⊕⊝	Consistent effect across trials
Hyperkalemia (K⁺ ≥5.5 mmol/L)	23 per 1,000	65 per 1,000 (35-120 per 1,000)	RR 2.87 (1.61, 5.11)	1,318 (three RCTs)	Moderate ⊕⊕⊕⊝	Downgraded for imprecision (few events, wide CI). Most cases mild and manageable
Any adverse event	500 per 1,000	525 per 1,000 (475-580)	RR 1.05 (0.95-1.16)	1,318 (three RCTs)	High ⊕⊕⊕⊕	No safety signal versus placebo

Discussion

Summary of Principal Findings

This systematic review and meta-analysis synthesizes current randomized trial evidence on baxdrostat for the treatment of RH. Results demonstrate, with moderate certainty, that adding baxdrostat to multidrug background regimens leads to statistically significant and clinically meaningful reductions in both systolic (approximately 8 mmHg) and diastolic (approximately 4 mmHg) blood pressure. The treatment effect appears dose-dependent, with greater reductions observed at 2 mg daily. From a safety perspective, baxdrostat is generally well tolerated, with an overall adverse event incidence comparable to placebo. The primary safety concern is a nearly threefold increased risk of hyperkalemia, a known and predictable effect of therapies that interfere with the RAAS. The absolute incidence remains modest, and reported cases were typically mild and manageable without requiring treatment discontinuation.

Recent Developments and Updated Evidence

Since the completion of our primary literature search, the BaxHTN Phase 3 trial has been published in the *New England Journal of Medicine* (October 2025) [27]. This publication confirms and strengthens our meta-analytic findings, reporting seated SBP reductions of -12.4 mmHg and -11.3 mmHg with baxdrostat 2 mg and 1 mg, respectively, versus placebo at 12 weeks (both p < 0.001). Ambulatory SBP reductions were similarly robust (-10.7 mmHg and -9.9 mmHg for 2 mg and 1 mg doses, respectively). These results align closely with our pooled estimates and further validate baxdrostat’s efficacy profile.

Additionally, results from the Bax24 Phase 3 trial were presented at the American Heart Association Scientific Sessions in November 2025 [35]. This trial evaluated baxdrostat in a broader population, including patients with uncontrolled hypertension on two or more medications. While a detailed peer-reviewed publication is pending, preliminary results reportedly showed significant blood pressure reductions consistent with previous trials. Upon peer-reviewed publication, Bax24 data should be incorporated into future meta-analyses to provide even more robust estimates of baxdrostat’s efficacy across the spectrum of hypertension.

Comparison With Other Therapies and Contextualization

Findings for baxdrostat are consistent with emerging data for other novel selective aldosterone synthase inhibitors. Lorundrostat, another selective aldosterone synthase inhibitor, has demonstrated significant blood pressure-lowering efficacy in Phase 2 and 3 trials [36]. A separate meta-analysis of Phase 2 trials of various aldosterone synthase inhibitors reported a similar magnitude of SBP reduction (pooled MD: -6.3 mmHg) and comparable hyperkalemia risk (pooled RR: 2.5) [37]. This consistency across different molecules strongly suggests a robust class effect for selective aldosterone synthase inhibitors, validating the therapeutic concept of selective aldosterone synthase inhibition and strengthening the rationale for considering this class in future clinical guidelines, pending confirmation of long-term safety and efficacy.

When contextualized against the current standard of care with MRAs, baxdrostat offers a distinct mechanistic advantage. While no head-to-head trials were included in this review, the magnitude of blood pressure reduction with baxdrostat appears similar to that achieved with spironolactone in the landmark PATHWAY-2 trial [14]. The key differentiator is baxdrostat’s highly selective, cortisol-sparing mechanism, which avoids the antiandrogenic and progestogenic side effects that plague spironolactone and often lead to treatment nonadherence.

The advent of selective aldosterone synthase inhibitors prompts a reevaluation of therapeutic goals in aldosterone-mediated hypertension. The objective shifts from simply blocking aldosterone effects to precisely normalizing its pathological overproduction while leaving other crucial hormonal pathways undisturbed. By selectively targeting the ligand (aldosterone) rather than the receptor, baxdrostat may preserve essential physiological mineralocorticoid receptor functions mediated by cortisol in nonepithelial tissues [24,25]. This positions aldosterone synthase inhibitors as potentially superior and more physiologically refined alternatives, particularly for patients with side effect intolerance or where precision targeting of the aldosterone pathway is paramount.

Explaining Heterogeneity and Contradictory Findings

Meta-analysis for SBP revealed substantial statistical heterogeneity (I² = 84%), largely driven by the HALO trial (NCT05137002) null result. Several plausible factors may account for this discrepancy. The HALO trial [28] utilized a shorter eight-week primary follow-up compared with 12 weeks in the positive BrigHTN trial [22] and BaxHTN trial [27]. This shorter time frame may have been insufficient to capture baxdrostat’s full antihypertensive time course.

Furthermore, HALO investigators noted potential suboptimal medication adherence within their study population, which can significantly attenuate treatment effects in placebo-controlled designs. Moderate to high heterogeneity observed in SBP outcomes likely reflects differences in follow-up duration, trial phase, and study conduct. The HALO trial evaluated outcomes at eight weeks, whereas BrigHTN and BaxHTN assessed endpoints at 12 weeks.

Exploratory sensitivity analyses suggest that exclusion of HALO reduces heterogeneity and increases pooled efficacy estimates, indicating that this study represents the principal source of between-study variability. These sensitivity findings should be interpreted cautiously, given the limited number of included trials. The subsequent success of the much larger and methodologically robust BaxHTN Phase 3 trial [27] strongly suggests that HALO findings may not reflect baxdrostat’s true efficacy. This conclusion is supported by our meta-analysis, in which positive, higher-quality trials dominate the pooled effect estimate. The shorter eight-week follow-up in HALO, compared with the 12-week endpoints in BrigHTN and BaxHTN, may have limited the full expression of the pharmacodynamic effect and contributed to its neutral statistical outcome.

Clinical Implications

For clinical practice, baxdrostat represents a significant addition to the limited armamentarium for managing RH. It can be positioned as a primary alternative to spironolactone for patients experiencing, or concerned about, its hormonal side effects. The predictable and manageable risk of hyperkalemia necessitates routine electrolyte monitoring, a practice already standard for patients receiving other RAAS-interfering therapies [38,39]. The development of baxdrostat introduces a truly novel mechanism to a therapeutic area that has seen little innovation for over two decades, directly addressing the critical unmet need of aldosterone dysregulation in high-risk hypertensive populations [40].

Research Implications

These findings highlight several key priorities for future research. The most critical need is data from large-scale, long-term cardiovascular outcome trials. Ongoing studies are designed to determine whether the observed blood pressure-lowering effects translate into tangible reductions in major adverse cardiovascular events, heart failure hospitalizations, and chronic kidney disease progression.

Second, head-to-head RCTs directly comparing baxdrostat with spironolactone are essential. Such trials would provide definitive evidence on comparative efficacy, tolerability, and impact on patient adherence and quality of life, information that is invaluable for guiding clinical decision-making.

Finally, further investigation is warranted in specific high-risk populations in which aldosterone excess is particularly prevalent and pathogenic, including patients with diagnosed primary aldosteronism, moderate-to-severe chronic kidney disease, and heart failure with preserved ejection fraction [41,42].

Strengths and Limitations

Primary strengths of this review include adherence to a preregistered protocol, a comprehensive unrestricted search strategy, rigorous methodology following PRISMA 2020 guidelines, and use of state-of-the-art tools for assessing risk of bias (RoB 2) and certainty of evidence (GRADE). To our knowledge, this represents the most current and comprehensive synthesis of RCT evidence for baxdrostat in RH.

However, several limitations must be acknowledged. The small number of included studies (three) inherently restricts the statistical power of subgroup analyses and precludes a robust assessment of publication bias. Substantial heterogeneity, primarily driven by one trial with differing design, warrants caution in interpreting the precise pooled effect size. Additionally, included trials focused on the surrogate endpoint of blood pressure over relatively short follow-up periods. Crucial data on long-term effects on major cardiovascular and renal outcomes are not yet available. Assessment of publication bias was limited by the small number of included trials; formal evaluation using funnel plots or regression-based methods was not performed, as such analyses are unreliable with fewer than ten studies. Although comprehensive database and registry searches were conducted, the possibility of unpublished or inaccessible data cannot be entirely excluded.

## Conclusions

Based on a meta-analysis of high-quality RCTs, baxdrostat, when added to standard background therapy, provides clinically significant blood pressure reductions in patients with RH. Its innovative, highly selective, cortisol-sparing mechanism results in a favorable overall safety profile, with the main risk being predictable, mild hyperkalemia that is generally manageable. Baxdrostat demonstrates clinically meaningful blood pressure reductions with a favorable safety profile; however, long-term cardiovascular outcome trials are needed to confirm whether these reductions translate into decreased cardiovascular events and improved patient outcomes.

## Appendices

Appendix A 

**Table 4:** Database search strategies

Database	Search strategy
PubMed	(“Baxdrostat” OR “CIN-107”) AND (“Resistant Hypertension” OR “Treatment-Resistant Hypertension”) AND (“Randomized Controlled Trial” OR RCT)
Embase	1. ('baxdrostat'/exp OR 'cin-107':ab,ti OR 'baxdrostat':ab,ti) 2. ('resistant hypertension'/exp OR 'treatment-resistant hypertension':ab,ti OR 'refractory hypertension':ab,ti) 3. ('randomized controlled trial'/exp OR 'randomized':ab,ti OR 'randomised':ab,ti OR 'rct':ab,ti) 4. 1 AND 2 AND 3
Cochrane CENTRAL	("Baxdrostat":ti,ab,kw OR "CIN-107":ti,ab,kw) AND ("resistant hypertension":ti,ab,kw OR "treatment-resistant hypertension":ti,ab,kw)
Scopus	TITLE-ABS-KEY("Baxdrostat" OR "CIN-107") AND TITLE-ABS-KEY("resistant hypertension" OR "treatment-resistant hypertension" OR "refractory hypertension") AND TITLE-ABS-KEY("randomized" OR "randomised" OR "RCT")
